# A multilevel transition perspective on embedding intersectoral action in local health policies

**DOI:** 10.1093/heapro/daaa131

**Published:** 2020-12-10

**Authors:** Sabina Super, Laurens W A Klerkx, Niels Hermens, Maria A Koelen

**Affiliations:** 1 Health and Society, Social Sciences Group, Wageningen University, Wageningen, The Netherlands; 2 Knowledge, Technology and Innovation Group, Wageningen University, Wageningen, The Netherlands; 3 Verwey-Jonker Institute, Utrecht, The Netherlands; 4 Health and Society, Social Sciences Group, Wageningen University, Wageningen, The Netherlands

**Keywords:** intersectoral action, Boundary spanner, social innovation, policy strategy, public health system transition

## Abstract

Intersectoral action is advocated as a social practice that can effectively address health inequalities and related social issues. Existing knowledge provides insight into factors that may facilitate or hinder successful intersectoral action, but not much is known about how intersectoral action evolves and becomes embedded in local health policies. This is where this study aims to make its contribution, by adopting the multilevel perspective on transitions, which is increasingly used to study social innovation in sustainability transitions but has not yet been applied to public health and health promotion. Through this perspective, it was unravelled how intersectoral action between youth-care organizations and community sports clubs became embedded in local health policies of Rotterdam, a large city in the Netherlands. A single explorative case study was conducted based on content analysis of policy documents and 15 in-depth interviews with policy officers, managers and field workers operating in the fields of youth and sports in Rotterdam. The findings showed that intersectoral action between community organizations and policymakers evolves through congruent processes at different levels that changed institutional logics. Moreover, it emerged that policymakers and other actors that advocate novel social practices and act as boundary spanners can adopt multiple strategies to embed these practices in local health policy. The multi-level perspective adds value to earlier approaches to research intersectoral collaboration for health promotion as it allows to better capture the politics involved in the social innovation processes. However, further sharpening and more comprehensive application of transition concepts to study transitions in public health and health promotion is needed.

## INTRODUCTION

Governments in developed countries are increasingly concerned with reducing health inequalities and improving health and well-being of vulnerable and socially disadvantaged groups (Graham, 2004). In tackling these health inequalities, policymakers are facing complex issues, recognizing that effective policy needs to address the underlying social determinants of health inequalities and that transforming social conditions cannot be done by governments alone (Irwin *et al*., 2006); for example, because many determinants of health, such as educational attainment, family income and social environment, cannot be addressed by public health organizations alone (Marmot *et al*., 2012). In face of increasing healthcare costs and cuts in health and social care budgets, new ways of addressing health inequalities and other social problems are sought (Borzaga and Fazzi, 2014; Westley *et al*., 2014) and social innovation is promoted, defined as ‘a collective process of learning involving civil society actors aimed to solve a societal need through changes in social practices’ (Edwards-Schachter and Wallace, 2017).

Intersectoral action has been advocated to address social issues and health inequalities effectively and to support social innovation (Corbin, 2017; Pedersen *et al*., 2017). In these collaborations, non-state welfare services and third-sector parties, such as community organizations, are taking a growing role (Milbourne, 2009), as they are considered to be able to improve the efficiency of the healthcare system especially when welfare states are under pressure from financial crises (Borzaga and Fazzi, 2014). In addition, these third sector parties are needed to effectively address the social determinants of health that are often outside the traditional health system. And finally, they have expertise in reaching and working with vulnerable groups (Milbourne, 2009) and possess the local knowledge that is needed to fit new ideas within a local context (Frantzeskaki *et al*., 2016). Hence, it is advocated that intersectoral action is needed to effectively address current complex and multi-faceted health issues (Roussos and Fawcett, 2000). However, intersectoral action is not self-evident and easy, because it needs to fit with multiple sectors’ aims and cultures and because participants have to get used to new relationships, procedures and structures (Koelen *et al*., 2012). In addition, public health is often not the core business of the involved third-sector parties and, therefore, they do not necessarily feel responsible or capable to work on public health issues. To deal with the difficulties in intersectoral action, scholars have developed frameworks that provide insights into the process of intersectoral action and how to manage it (Koelen *et al.*, 2012; Corbin, 2017; Hermens *et al*., 2017). These frameworks include information about (i) how the institutional and personal background of the organizations and individuals participating can influence the intersectoral collaboration and its outcomes, and (ii) preconditions for successful intersectoral collaboration, such as a boundary spanning leadership style and a shared vision. However, the frameworks on intersectoral action and social innovation rarely unravel the deeper dynamics behind the evolvement of intersectoral action and how it becomes embedded in local policies. This is where this study aims to make its contribution, by analysing the evolution and embedding of intersectoral action between youth-care organizations and sports clubs in local policy as a transition process. Rotmans *et al.* have defined a transition process as ‘a set of connected changes, which reinforce each other but take place in several different areas, such as technology, the economy, institutions, behaviour, culture, ecology and belief systems’ [(Rotmans *et al*., 2001), p. 16]. In line with this we consider the development of intersectoral action between youth-care organizations and sports clubs to be a transition process because this process denotes a comprehensive institutional, cultural, behavioural and economic change from one dynamic equilibrium to another (Rotmans *et al.*, 2001). This new equilibrium is characterized by youth-care organizations and sports clubs working more integrally on a shared issue, are held collectively responsible for policy outcomes (e.g. reducing youth care costs), and share resources such as policy budgets to address social issues, rather than working separately from one another. Second, we consider this to be a transition process because new parties become involved in the public health domain that have not previously been involved and that do not have public health related aims in their core business. To analyse this transition process, we adopted Geels’ (Geels, 2002) multilevel perspective on transitions. This perspective is increasingly used to analyse transitions in social, public health and healthcare systems (Johansen and van den Bosch, 2017; Hassink *et al*., 2018; Frantzeskaki and Wittmayer, 2019; Köhler *et al*., 2019) as it offers a valuable perspective on how social innovations are dynamically shaped and permeate existing policies and ways of doing. The multilevel perspective on transitions goes beyond current perspectives on intersectoral action by offering more in-depth understanding of the dynamics of evolving systems, by seeing transition as a long-term process with co-evolving changes at multiple levels and as a political processes in which actors utilize specific strategies to change the system.

To unravel the dynamics of the evolution and embedding of intersectoral action in local policies, we conducted an explorative single case study on how intersectoral action between youth-care organizations and community sports clubs became institutionalized in local policies of Rotterdam, a large city in the Netherlands. The embedding of intersectoral in local policies is a process that started two decades ago and this currently still ongoing. Youth-care organizations in the Netherlands provide services to youths who are experiencing problems in their personal development, for example because of learning or behavioural problems or because their parents are incapable of providing proper care. In light of decreasing care budgets, policymakers, researchers and field workers increasingly recognize intersectoral action between youth-care organizations and community sports clubs as a means to improve the physical, mental and emotional health of vulnerable youths (Hermens *et al.*, 2017; Super *et al*., 2018). The underlying assumption is that sports participation can strengthen the personal development of vulnerable groups, by providing a safe and supportive climate in which new life skills, competencies and values can be learned that are valuable for everyday life. As such, sports participation can complement the care youths receive from care organizations in reaching positive (health) outcomes.

In the next section we will further outline the analytical approach, followed by methods and results. The paper ends with a discussion on the implications of our findings for debates on social innovation and intersectoral collaboration.

### Analytical approach: the multilevel perspective on transitions

Central to the multilevel perspective on transitions are phenomena at three analytical levels, i.e. regime, landscape and niches (Geels, 2002). The *regime* level, which is the core level, can be defined as a set of historically established and institutionalized rules and beliefs that guide thoughts and behaviours of actors in a certain societal system, such as the youth-care and the sports sector. These so-called institutional logics (Fuenfschilling and Truffer, 2014; Smink *et al*., 2015), defined by Thornton and Ocasio as ‘the socially constructed, historical patterns of material practices, assumptions, values, beliefs, and rules by which individuals produce and reproduce their material subsistence, organize time and space, and provide meaning to their social reality’ [(Thornton and Ocasio, 1999), p. 804] may hinder the institutionalization of novel practices. The *landscape* level refers to structural macro-level trends and changes external to regimes (Geels, 2002), such as the international economic situation, public awareness and major governmental ideas. One element of the landscape level is a so-called game changer, described by Loorbach *et al.* (Loorbach *et al*., 2016) as a macrophenomenon that pushes a complex societal system out of its dynamic equilibrium. A game changing event, such as an economic crisis, causes tensions leading to changes in the institutionalized rules and beliefs and hence to increased legitimacy for new ways of working. *Niches* can be defined as settings where organizations and individuals develop, test, broaden and refine new ways of working (Geels, 2002). Niches go beyond experiments by single entrepreneurs and embrace learning processes and network-building activities that may slowly influence dominant rules and beliefs (Raven and Verbong, 2007). Niches may gain a momentum that leads to their becoming embedded in national or local policies, for example when game-changers cause large tensions within a regime (Loorbach *et al*. 2017).

The driver for transition is the change agency by particular actors within niches and regimes (Farla *et al*., 2012). Different terms have been used for these actors, such as boundary spanners (Smink *et al.*, 2015), institutional entrepreneurs (Pacheco *et al*., 2010) and hybrid actors who sit between niche and regime (Elzen *et al*., 2012). Because all these types of actors share boundary spanning characteristics, the term *boundary spanner* is used in this article. Boundary spanners can adopt multiple strategies to drive transitions, for example framing-specific practices as solutions for societal problems and building networks and advocacy coalitions around novel practices (Pacheco *et al.*, 2010; Westley *et al*., 2013). In transitions involving multiple regimes, boundary spanners need to bridge the gap between representatives from the different sectors, for example through formulating shared ideas, visions, and goals and building trust (Hassink *et al.*, 2018). The multilevel perspective on transitions will be adopted in this study to demonstrate the dynamic evolvement and embedding of the intersectoral action between youth-care organizations and local sports clubs in local health policies.

## METHODS

We conducted a single case study based on content analysis of policy documents and in-depth interviews with policy officers, managers and field workers operating in the fields of youth care and sports in Rotterdam. Single case studies help to obtain the detailed and contextualized knowledge needed to unravel complex processes that take place in real-life situations (Flyvbjerg, 2006). Following earlier studies on social transitions (e.g. Johansen and van den Bosch, 2017; Hassink *et al.*, 2018), we adopted a timelining method to distinguish the processes and events in the evolution of intersectoral action between youth-care organizations and sports clubs in Rotterdam and its embedding in Rotterdam social policies.

### Data collection

Rotterdam youth and sports policy documents (*n* = 13) were gathered via civil servants in the municipality of Rotterdam. We chose to gather policy documents released between 1990 and 2016, because the wider social role of sports attracted attention in Dutch national and local policies from the end of the twentieth century (Leenaars *et al*., 2016). Individual in-depth interviews were conducted with 15 policy officers, managers and field workers who played a role in the youth-care and sports sectors in Rotterdam between 2000 and 2016. Interviewees were selected through a snowball procedure. Seven interviewees represented the Rotterdam sports regime, five interviewees represented the Rotterdam youth-care regime and three interviewees were niche actors from the Rotterdam sports (*n* = 1), youth care (*n* = 1) and social (*n* = 1) sectors who set up youth sports projects from the grassroots. Loorbach *et al.* have recognized that some actors might be more related (Loorbach *et al.*, 2017) to a regime contexts and others to a niche context, and even other individuals are more flexible in relating themselves to both niches and regimes (Elzen *et al.*, 2012). We have labelled the interviewees to belong to a regime or a niche based on their role in the transition towards intersectoral embedding, where actors were seen as being regime actors when they were part of the predominant institutional logics and infrastructures (e.g. youth policy worker), and were seen as being niche actors when they were involved in developing new innovative ways of working (e.g. sports professional in a grassroots project).

The interviews were conducted by the first and the second author and were guided by a timeline on a sheet of A4-size paper. Interviewees were asked to mark periods and moments that were important for how intersectoral action between youth-care organizations and sports clubs evolved in Rotterdam, and for how it became embedded in local policies. For each period and moment, the interviewer asked questions regarding its cause and impact and about the role played by different actors. All interviews were audiotaped and transcribed verbatim style. The interviews lasted 45–75 min. All interviewees gave informed consent on the understanding that the interviews would be tape recorded and that their anonymity would be guaranteed.

### Analysis

The data were analysed in three steps. First, text segments were coded about sports in the youth policy documents and text segments about social issues, including youth care and health issues, in the sports policy documents. Using this information, the changes in main ideas about the wider social role for sports in Rotterdam youth care and sports policies were plotted against a timeline, which was used as background information during the interviews and for interpreting the interview data. Second, text segments were coded in the interviews in which interviewees addressed niche level developments, regime changes and landscape developments. These developments were coded using the definitions given in the ‘Analytical Approach’ section (see above). Text segments were also coded in which interviewees addressed how the sports projects and intersectoral action at the niche level became embedded in Rotterdam local policies. Third, the coded text segments were placed in chronological order. Two of the authors discussed these text segments and expanded the initial timeline with the information about the processes and events that were important for how the intersectoral action evolved and became embedded in Rotterdam policies.

## FINDINGS

**Fig. 1:**
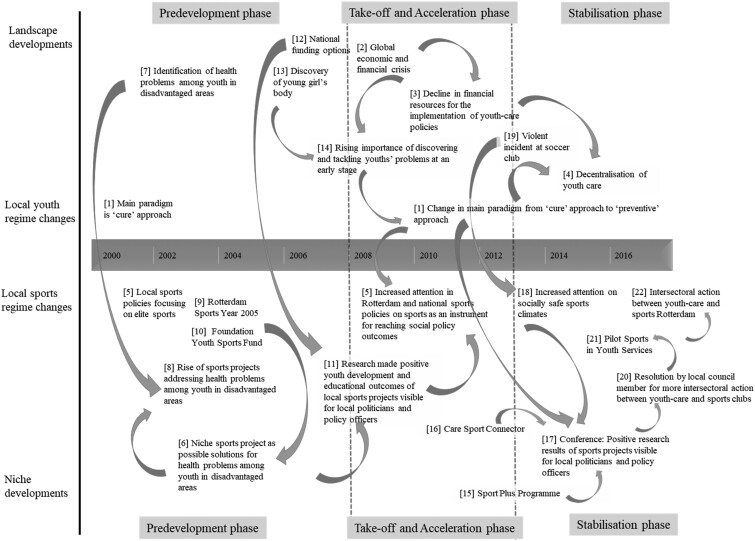
Overview of the evolution and embedding of intersectoral action between youth care and sports in Rotterdam.

The results are divided in two sections. In the first section, the main trends in Rotterdam sports and youth-care regimes between 1990 and 2016 according to the policy documents are described. In the second section, using the interview data, we describe how intersectoral action between youth-care organizations and sports clubs evolved in Rotterdam and how it became embedded in Rotterdam social policy. The second section is divided into subsections that align with the phases through which transitions go (Rotmans *et al.*, 2001): a predevelopment phase, a take-off phase, an acceleration phase and a stabilization phase. As we observed a large overlap in the processes taking place in the take-off and acceleration phases, these two are described in one subsection. Figure 1 summarizes the evolution and the embedding of intersectoral action between youth-care and sports organizations in Rotterdam. The numbers between brackets in the text below, refer to the numbered events and developments in Figure 1.

### Trends in Rotterdam youth-care and sports regimes

Starting around 2005, the main paradigm of youth care services slowly changed from a ‘curative’ approach, fusing on solving problems faced by socially vulnerable youths, towards a ‘preventive’ approach, focusing on discovering and tackling youths’ problems at an early stage [1]. A youth policy document from 2007, for example, encouraged youth professionals to exchange information with community organizations to be able to signal problems early. In 2008, the financial crisis, which was a game-changing event at the landscape level [2], caused large tensions in the Dutch youth-care regime, requiring youth-care regime actors to seek more efficient ways to address youth issues [3]. For example, the Dutch national government tried to improve the sector’s efficiency by decentralizing the responsibility for youth care from the national government to the local governments in January 2015 [4]. This move was grounded in the belief that local policymakers are more familiar with the local context where youths grow up and hence can more adequately and effectively address the youths’ and their families’ problems. Along with this decentralization of responsibilities, the Dutch national and local governments increasingly expected youth-care organizations to encourage youths and their families to ask for support from their family, friends, neighbours and community organizations. This development at the landscape level further pressured changes in the local youth-care regime in Rotterdam. Since 2015, the Rotterdam youth-care regime has increasingly assigned community organizations a role as a pedagogical setting, thereby acknowledging the potential role of sports in reaching educational and developmental outcomes.

The sports regime underwent a comparable change in paradigm, changing from a focus on elite sport (around 1991), focusing on recognizing and supporting talented sportsmen to become professional athletes, towards a focus on increasing sports participation rates in the general population and specifically of vulnerable groups, acknowledging the wider social role of sports (around 2009) [5]. This change of paradigm within the Rotterdam sports policies was reported to be one of the reasons for founding a local organization (i.e. Rotterdam Sportsupport) that aimed to increase sports participation among diverse groups in Rotterdam, such as socially vulnerable youth, long-term unemployed people and older people. The underlying assumption was that participating in sports facilitates positive development in these groups. The most recent Rotterdam sports policy document, published in 2016, included the aim that sports organizations should become structural partners for public health organizations.

### How intersectoral action between youth care and sports evolved

#### Predevelopment phase (2003–2008)

Between 2003 and 2008, numerous sports projects addressing youth health issues were developed in Rotterdam, which many of the interviewees defined as niche activities of intersectoral action between youth-care organizations and sports clubs [6]. According to the interviewees, the early niche activities gained momentum when a sense of urgency was felt by youth organizations that were confronted with health-related issues relating to an increase in overweight and a decline in physical activity and sports participation rates among youth in the city’s socially disadvantaged areas [7]. The interviewees reported that regime actors sympathetic towards these sports projects framed them as solutions for these health-related youth-care issues [8], and this persuaded policymakers to increase the financial resources available for developing and implementing youth sports projects. At the same time on regime level, two developments led to an increased availability of resources for vulnerable groups to participate in sports. First, resources became available for novel sports programmes because of the Rotterdam Sports Year 2005 [9]. Second, the foundation of the Rotterdam Youth Sports Fund created possibilities for low income families to acquire resources for sports club membership [10].

On a regime level, the role of sports clubs in reaching wider social outcomes gained attention since 2007 as well. Research on sport programmes showed that they led not only to improvements in physical health but also to social and educational outcomes [11]. Actors sympathetic towards the niche sports activities took advantage of this to create more legitimacy for sports projects addressing youth developments issues. The increased attention on regime level on the wider social role of sports coincided with rising national funding opportunities for developing and implementing sports projects at local level [12]. One national programme funded the Sports Care Tracks, in which youth-care professionals enrolled socially vulnerable youths in sports clubs and exchanged information with sports coaches about the youths’ personal development. According to the interviewees, the Sports Care Tracks were the first activities in which youth-care organizations and sports clubs collaborated, creating visibility at the landscape level. The interviewees also reported one game-changing event at the landscape level as being of particular importance for increasing the legitimacy of intersectoral action: the discovery of a young girl’s body in a river in Rotterdam as a result of intra-family violence [13]. This signalled the urgency for improved information exchange between youth-care organizations and voluntary community organizations, on the assumption that this creates possibilities to tackle problematic family situations at an early stage [14].

A development that the interviewees reported as pivotal for the evolution of intersectoral action between youth-care and sport organizations, which was not linked to the increase in national and local resources, was a niche project involving a sports pedagogue being employed at a local sports club [6]. This niche project started when an employee from a housing corporation identified the fact that many youths in a socially disadvantaged area in Rotterdam lacked a supportive social environment at home. As the employee believed that sports clubs could provide youths with an additional supportive environment, the housing corporation recruited a so-called sports pedagogue who supported sports coaches at a sports club in the Rotterdam area in creating a socially safe and supportive sports climate. The interviewees felt that this niche project formed the basis for the social transition to intersectoral action between youth-care organizations and sports clubs.

#### Take-off and acceleration phase (2008–2013)

Between 2008 and 2013, intersectoral action between youth-care and sports clubs rapidly gained legitimacy in Rotterdam youth-care and sports regimes. The interviews revealed that this was triggered by the complex interaction of processes at the landscape level and at the niche level. Due to pressures from the landscape level (e.g. financial crisis) [2], youth-care organizations were forced to start collaborating with community organizations, such as sports clubs, to be able to address health issues more efficiently. This was reported to be crucial because it created a political process in which different actors were competing for resources from the same policy budgets [3]. For example, one interviewee explained that disseminating positive social outcomes of existing niche sports projects [11] and framing sports projects as efficient ways to address social issues [5] were used as strategies to create changes in institutional logics at the regime level and hence obtain new resources from local policy: ‘*We knew that presenting a research report about successful sports projects a couple of weeks before the local policymakers were deciding about how to divide the sports policy budget had helped to gain support for existing and new sports projects addressing youth issues.*’ The interviewees reported that this was an important step in the social transition to intersectoral action because it expanded existing collaborations to different parts of the city.

Another event that was reported as crucial for the evolution and embedding of intersectoral action was the development of the Sports Plus Programme [15]. This programme, initiated by Rotterdam Sportsupport, had the aim of encouraging sports clubs and social sector organizations to jointly generate and implement sports projects that address social issues. Many of the collaborations that emerged in the context of the Sport Plus Programme were niche initiatives from sports club volunteers and/or youth-care professionals, but the programme also provided opportunities for networking activities with powerful regime actors, such as managers of youth-care organizations and local policymakers. According to the interviewees, these networking activities were crucial for the evolution and embedding of intersectoral action between youth care and sports in Rotterdam.

Besides opportunities for intersectoral action arising from the sports policy plans, the interviewees also reported that the Care Sport Connector role was crucial in creating new niche level collaborations and in expanding existing ones [16]. Care Sport Connectors were appointed, following a national policy plan to increase sports participation in the Netherlands and particularly of socially vulnerable people. The interviewees defined the work of the Care Sports Connector as one of the first institutionalized forms of intersectoral action between youth-care organizations and sports clubs in Rotterdam. A final event on the niche level that the interviewees reported as central in the acceleration phase was a conference organized in 2013 [17]. Whereas Rotterdam Sportsupport initially organized this conference to gain legitimacy for pedagogical support for sports clubs by presenting positive findings from a study on the sports pedagogue’s work, the conference became relevant for youth care and sports regime actors when it was linked to two landscape developments. To youth-care sector representatives, the conference was framed as a setting where they could obtain information about how to put the preventive paradigm into practice. A youth policymaker explained how this persuaded him to attend the conference and to embed collaboration with sports organizations in youth policy plans: ‘*For me, the most crucial moment was the conference [in 2013]. In conjunction with us preparing the upcoming reforms in youth policies, it made me more open to intersectoral action with sports clubs.*’ To sports sector representatives, the conference was framed as a setting where they could obtain information about how a sports pedagogue could support them in creating a socially safe sports climate [18], which was perceived as particularly urgent at that time because of a violent incident during a soccer match in another Dutch city in 2012 during which an assistant referee was beaten to death (i.e. a game changer at landscape level) [19]. The ideas presented at the conference prompted a local council member to submit a resolution that youth-care organizations should support sports clubs in creating socially safer sports climates [20]. According to the interviewees, this resolution started the embedding of intersectoral action between youth-care and sports clubs in Rotterdam local policy.

#### Stabilization phase (2014–2016)

The stabilization phase started in 2014 with a pilot project called Sports in Youth Services in one of the city’s 12 areas [21]. Whereas before 2014 the collaborative actions between youth-care organizations and sports clubs were built around often short-term niche sports projects, Sports in Youth Services had the aim of creating structural networks of youth-care organizations and sports clubs. The partners in the pilot network explored how they could jointly increase socially vulnerable youths’ sports participation and improve the sports clubs’ socio-pedagogical climate. In addition, they tried to develop and implement sports programmes serving socially vulnerable youth. To support the pilot, the sports and youth aldermen and the managers of the organizations participating in the pilot network signed an agreement in which they committed themselves to the network. According to the interviewees, this regime support was required to create legitimacy for the intersectoral action among the participating professionals and volunteers.

In 2016, Sport in Youth Services networks were formed in all 12 areas of Rotterdam [22]. The success of building this network of sports projects was, according to the interviewees, mainly due to the capabilities of the leader of the pilot network in managing the differences in institutional backgrounds between youth-care professionals and unpaid volunteers from sports clubs. Appointing a youth policymaker as pilot project leader had helped to embed the intersectoral action in novel youth policies, for example because the pilot leader could frame it in such ways that it fitted with the ideas in novel youth policy plans. ‘*I knew that [to embed the intersectoral action in youth policy plans] such a pilot needs to link with the youth policy’s bigger picture. So, when the new youth policy document indicated that intersectoral action is needed for positive youth development, I started negotiating that Sport in Youth Services should be part of the novel youth policy.*’ In this way, the project Sport in Youth Services became part of the institutional logics of the youth and sport regime.

## DISCUSSION

By adopting a multilevel perspective on transitions, this study aimed to explore how the transition towards the embedding of intersectoral action between youth-care organizations and local sports clubs evolved through congruent processes of social innovation at different levels. Previous studies have demonstrated that intersectoral collaboration is not self-evident, as many hurdles may need to be taken for successful collaboration, such as creating a shared interest and language between groups of professionals (Lasker *et al*., 2001; Koelen *et al.*, 2012; Hermens *et al.*, 2017). A study conducted on intersectoral action between youth care organizations and sports organizations in the Netherlands revealed that different factors served as facilitators or barriers in different stages of the coordinated action, such as a positive attitude towards collaboration and individual competences in collaborating with different organizations (Hermens *et al.*, 2017). In addition, the success of the coordinated action appeared to be strongly depended on personal factors such as pre-existing personal relationships between actors, attitudes of actors towards collaboration and knowledge about for example implementation possibilities. To address these hurdles in intersectoral collaboration, existing research brought about frameworks that include information on preconditions for the development and continuation of intersectoral action (Hanleybrown *et al*., 2012; Koelen *et al.*, 2012; Flood *et al*., 2015; Corbin, 2017). For example, Koelen *et al.* distinguished three clusters of preconditions for intersectoral action (Koelen *et al.*, 2012), i.e. institutional elements, personal elements and organizational elements. This current study complements earlier work by demonstrating the dynamics behind the evolution and embedding of intersectoral action in local health policies. In addition, the multilevel perspective on transitions allowed for a better understanding of the complexity of the development towards intersectoral collaboration and the roles that actors have taken in the transition, as it highlights the political nature of social transitions and the strategic roles that actors can play in this process. As pointed out by Grin and Broerse transitions ‘can neither be controlled nor steered, but they can be influenced in their direction and speed [_**…**_] through the guiding vision of sustainable development and joint learning among multiple stakeholders, comprising a cyclical process of development at different levels and in different domains’ [(Grin and Broerse, 2017), p. 13]. Our study highlights the political nature of social innovation, in which conflict and power differences play an important role.

Adopting the multilevel perspective helped to reveal that social innovations underpinning intersectoral collaboration are not only based on processes of collective learning (Edwards-Schachter and Wallace, 2017), but also that they include political processes in which *boundary spanning actors* try to change a regime’s institutional logics, strive for legitimacy and compete for resources from local policy budgets. For example, the conference organized in 2013 offered a unique opportunity to engage important stakeholders and to lobby for policy budgets by framing the conference’s aims differently for different stakeholders. The conference organizers reframed the results from the niche sports projects after a violent incident at a sports club, to highlight how these projects could help in addressing social issues in deprived neighbourhoods by creating a safe climate at local sports club through involving a sports pedagogue. This current study showed that boundary spanners used different strategies, such as framing, dissemination of positive findings and network building, to gain support for the evolution and embedding of intersectoral action between youth-care organizations and sports clubs, and hence for sparking a transition.

As regards *framing and dissemination of positive findings*, previous research has indicated that visibility is important for the success of intersectoral action, because visibility of activities and their outcomes can motivate participants to continue their collaborative work and because it allows them to gain political and financial support to continue a partnership (Koelen *et al.*, 2009). This study enriches Koelen *et al.*’s finding by showing that the visibility of intersectoral action is especially important for its evolution and embedding in local policy, and that visibility can be created by framing and dissemination strategies. It emerged that framing niche level intersectoral action as a solution for persistent problems at the landscape level can create a basis for its embedding in local policy. For example, most of the niche level intersectoral action between youth-care organizations and sports clubs evolved as a means to address persistent problems at the landscape level (i.e. increased overweight among youth, reduced social safety at community sports clubs and the decentralization in Dutch national youth policies that forced local policymakers to put a novel paradigm into practice). This indicates that social innovation for intersectoral collaboration also benefits from having a broader systemic awareness to make use of larger societal phenomena. However, as pointed out by Holt *et al.* reframing issues in terms of health in intersectoral (Holt *et al*., 2017) policymaking also runs the risk of developing a too narrow focus on health-related behaviours in small-scale interventions, thereby neglecting the broader social determinants of health and other welfare policies outside the health sector that are relevant for addressing health inequities. ‘*We caution that it is necessary to frame problems broadly in line with the wider structural social determinants of health and strike a balance between health outcomes and social or economic objectives* [in intersectoral policymaking]’ [(Holt *et al.*, 2017), p. 888]. So although framing positive outcomes of niche level sports projects as solutions for health-related issues at the landscape level can be useful for gaining legitimacy and support within the health sector, it remains a question whether this strategy also proves useful in addressing the underlying causes of health inequalities for socially vulnerable youth.

As regards *network building strategies*, this study showed that the niche sport projects offered an important platform for building and extending networks. As described in the introduction, niches are settings where organizations and individuals develop, test, broaden and refine new ways of working, including but not limited to building new alliances and partnerships (Geels, 2002). This current study showed that new actors became involved as pilot projects proved successful, for example in the case of the pilot Sport in Youth Services which was spread to all Rotterdam regions involving more stakeholders after initial success in one region. Previous studies on social transitions involving multiple regimes (Hassink *et al.*, 2018) have shown that successful boundary spanners need to be familiar with the institutional logics of the regimes of both sectors and need to speak the languages of both sectors because this helps to create trust relationships between actors from different sectors. A similar result was found in this study as several interviewees mentioned that the success of the pilot Sport in Youth Services was due to the fact that the project leader was able to manage differences in institutional logics, thereby being able to embed the pilot in youth policy plans. This study broadens existing knowledge on intersectoral collaboration for health promotion (Koelen *et al*., 2008, 2012) by indicating that the evolution and embedding of novel intersectoral action also requires boundary spanning leadership that creates connections between niche level activities and regimes and that can support the process of challenging, renegotiating, and reconciling the institutional logics of the different sectors, for example by tapping into landscape developments and reframing niche projects to align with these developments. Connecting multiple niches has been found to support learning processes in transitions (Smith *et al*., 2010), and the current study indicates that connecting niches can also help to gain legitimacy and critical mass and hence to spark changes in institutional logics.

### Limitations

Applying the multilevel perspective on transitions has helped us to unravel the overall process of how intersectoral action between youth-care organizations and sports clubs evolved and became embedded in local policy by showing that processes and actions at various levels interact to create a momentum for strengthening intersectoral collaboration. Even though the multilevel perspective is more and more applied to social innovations (Hassink *et al.*, 2018; Frantzeskaki and Wittmayer, 2019), there are also several criticisms that can be offered on the application of the multilevel perspective on transitions to this setting of multisectoral social innovation. First, this analytical perspective has been developed for studying socio-technical innovations, such as transitions in the energy or infrastructure system (Markard *et al*., 2012). These socio-technical transitions differ from social innovation towards intersectoral collaboration that has been the topic of this article, on a number of important characteristics. For example, the type of actors that are involved differ, where often large firms are involved in socio-technical innovations and small voluntary organizations were involved in this specific case of social innovation towards intersectoral collaboration. Therefore, different processes may be at play in socio-technical innovations than in social innovations and the multilevel perspective needs to be refined in order to capture these differences better (Markard *et al.*, 2012). A second criticism on using the multilevel perspective is that we found that it could not fully capture micro-level processes in transitions, such as how boundary spanning actors build relationships with powerful regime actor or what precise strategies forerunners use to develop and manage niche activities from the grassroots. This is an inherent trade-off recognized in transition studies (Farla *et al.*, 2012). A third criticism offered on the multilevel perspective on transition relates to the difficulties of operationalizing the different concepts, especially of the ‘regime’ which has been put synonymous with ‘system’ or with ‘rules and institutional logics’ (Holtz *et al*., 2008; Geels, 2011). It is for this reason that we have defined the various concepts in the theoretical framework and have coded various developments in the results section according to these definitions. However, we should be aware that different interpretations could be offered of the data when different definitions are applied. In addition, it is important to note that this study was explorative in nature and applied the multilevel model in a rather pragmatic way as an analytical heuristic. Therefore, different interpretations and reflections on using the concepts of the theoretical framework are possible, such as the clear analytical distinction we made between niche and regime actors. As our analysis also showed, and in line with arguments in the literature (Elzen *et al.*, 2012; Runhaar *et al*., 2020), the distinction between niche and regime actors and how they contribute to transitions (as either barriers to change or agents of change) is less clear cut in practice than in theory. Thus, in practice these roles and positions are not without contestations between the regime and niche actors, and rather are political concepts that define each’s role, responsibilities and opportunities within a social transition. Thus, further debate and scrutiny are needed about what constitutes niches and regimes in social transitions in multisectoral settings (following Sutherland *et al*., 2015; Hassink *et al.*, 2018) and how different actors can be positioned in a such a multisectoral social transition.

Unravelling a single case is strength and a limitation. On the one hand, it created a thorough understanding of the evolution and embedding of intersectoral action between youth-care organizations and sports clubs in one city. On the other hand, it may be hard to generalize the findings. Although landscape developments in different Dutch cities are likely to be similar, the transition revealed in this study may be limited to Rotterdam. It would therefore be interesting to replicate this study in other cities in The Netherlands or Western Europe, as this would enable comparison of processes in cities with and without such transitions under almost similar landscape conditions, thereby providing insights into the most crucial processes and strategies in social transitions and especially transitions to intersectoral action between youth-care and sports.

A second limitation of this case study concerns the fact that it zooms in on an episode of a transition which is still ongoing (i.e. a transition in the making) and has a longer history. We have analysed how intersectoral action between youth care organizations and sports clubs have started to become embedded in the youth care and sports regime, but at the same time we have to recognize that the data that are presented in this study cannot fully show the regime prior to 2003 (e.g. the systemic order of structures, cultures and practices that fundamentally needed to change) and the political contestation then about the origin, direction and type of the desired transition. It also describes only the first phase of this transition process and that future developments may facilitate or hinder this process towards the further embedding at the regime level. Embedding refers to a linking process between regime level and niches, that can lead to changes in the regime as well as in a niche (Elzen *et al.*, 2012). As part of this linking process, new ways of working that are developed at the niche level can become part of (i.e. become embedded at) the institutional logics at the regime level. An example of this embedding in the current case study is the inclusion of the aim that sports organizations should become structural partners for public health organizations in a sports policy document in 2016. Although becoming structural partners signals that new ways of working are becoming embedded in the institutional logics at regime level, this process is still evolving. As pointed out by Elzen *et al.* the linking process (or: embedding) is ‘the result of a continuous process of making and breaking new connections in which some connections may become stable while others are short-lived’ [(Elzen *et al.*, 2012), p. 3]. Therefore, future studies would be necessary to see how this process develops further and how the intersectoral action between youth care organizations and sports clubs will shape the institutional logics of the involved regimes in the next decades. A third limitation is the retrospective nature of the case study, where interviewees were asked to reflect on developments dating back sometimes more than 20 years ago. The timeline proved useful in placing developments and events in a chronological order, but the interviewees’ stories were undoubtedly coloured by recall bias. Interviewees, for example, may have failed to report on competing developments or conflicting interests by only recalling what the outcomes of the process were. Future studies would benefit from studying a case continuously and for a longer period of time, to observe how competing developments occur and either gain or lose interest and attention over time.

## CONCLUSION

Adopting a multilevel perspective on transitions to unravel how intersectoral action in the social and health policy field evolves and becomes embedded in local policy is to the best of our knowledge unique in the public health and health promotion field. This study was explorative in nature and demonstrated transition processes involved in the development towards intersectoral action between youth care and sports, through a mixture of landscape developments, niche actors developing small-scale novel practices from the grassroots and change agency by boundary spanners between niches and regimes sympathetic towards a novel social practice. The multi-level perspective adds value to earlier approaches to research intersectoral collaboration for health promotion as it allows to better capture the politics involved in the social innovation processes, highlighting the sorts of regime changes needed, the barriers encountered, the power dynamics at play and the influence and mobilization of external landscape type events and dynamics. However, further debate about the application of concepts from the multilevel perspective on transitions in public health and health promotion is needed, to more sharply define what constitutes a ‘full’ social transition, the role of multiple regimes in intersectoral social transitions, and to identify the role that various actors play in these transitions.

## FUNDING

The study was funded by NWO, the Dutch Organisation for Scientific Research (project number: 328-98-007).
